# Emergence of high drug resistant bacterial isolates from patients with health care associated infections at Jimma University medical center: a cross sectional study

**DOI:** 10.1186/s13756-018-0431-0

**Published:** 2018-11-19

**Authors:** Mulatu Gashaw, Melkamu Berhane, Sisay Bekele, Gebre Kibru, Lule Teshager, Yonas Yilma, Yesuf Ahmed, Netsanet Fentahun, Henok Assefa, Andreas Wieser, Esayas Kebede Gudina, Solomon Ali

**Affiliations:** 1 School of Medical Laboratory Science, Jimma University, Jimma, Ethiopia; 2 Department of Pediatrics and Child Health, Jimma University, Jimma, Ethiopia; 3 Department of Ophthalmology, Jimma University, Jimma, Ethiopia; 4 Department of Surgery, Jimma University, Jimma, Ethiopia; 5 Department of Obstetrics and Gynecology, Jimma University, Jimma, Ethiopia; 6 Department of Health Education and Behavioral Health, Jimma University, Jimma, Ethiopia; 7 Department of Epidemiology and Statistics, Jimma University, Jimma, Ethiopia; 8 Head of the parasitology laboratory and deputy head of the molecular diagnostics laboratory at the Max von Pettenkofer-Institute, Ludwigs-Maximilians-University (LMU), München, Germany; 9 Department of Internal Medicine, Jimma University, Jimma, Ethiopia; 10 WHO-TDR clinical research former fellow at AERAS Africa and Rockville, Rockville, MD USA; 11 Institute of Health, Jimma University, P.O. Box 1368, Jimma, Ethiopia

**Keywords:** Antimicrobial agents, Drug resistant isolates, Multidrug resistance, Extensively resistance, Pandrug resistance, Carbapenem resistance, Extended spectrum beta-lactamase

## Abstract

**Background:**

The rates of resistant microorganisms which complicate the management of healthcare associated infections (HAIs) are increasing worldwide and getting more serious in developing countries. The objective of this study was to describe microbiological features and resistance profiles of bacterial pathogens of HAIs in Jimma University Medical Center (JUMC) in Ethiopia.

**Methods:**

Institution based cross sectional study was carried out on hospitalized patients from May to September, 2016 in JUMC. Different clinical specimens were collected from patients who were suspected to hospital acquired infections. The specimens were processed to identify bacterial etiologies following standard microbiological methods. Antibacterial susceptibility was determined in vitro by Kirby-Bauer disk diffusion method following Clinical and Laboratory Standards Institute guidelines.

**Results:**

Overall, 126 bacterial etiologies were isolated from 118 patients who had HAIs. Of these, 100 (79.4%) were gram negative and the remaining were gram positive. The most common isolates were *Escherichia coli* 31(24.6%), *Klebsiella* species 30(23.8%) and *Staphylococcus aureus* 26 (20.6%). Of 126 bacterial isolates, 38 (30.2%), 52 (41.3%), and 24 (19%) were multidrug-resistant (MDR, resistant to at least one agent in three or more antimicrobial categories), extensively drug resistant (XDR, resistant to at least one agent in all but two or fewer antimicrobial categories (i.e. bacterial isolates remain susceptible to only one or two categories), pan-drug resistant (PDR, resistant to all antibiotic classes) respectively. More than half of isolated gram-negative rods (51%) were positive for extended spectrum beta-lactamase (ESBL) and/or AmpC; and 25% of gram negative isolates were also resistant to carbapenem antibiotics.

**Conclusions:**

The pattern of drug resistant bacteria in patients with healthcare associated infection at JUMC is alarming. This calls for coordinated efforts from all stakeholders to prevent HAIs and drug resistance in the study setting.

## Introduction

The emergence and rapid spread of multidrug resistant pathogenic bacteria is becoming a global health challenge [1]. Recent studies showed an increasing rate of bacterial resistance against available antibiotics. The problem is more pronounced in developing countries attributed to limited antibiotic option, irrational drug use, poor drug quality, poor sanitation, malnutrition, poor and inadequate health care systems, and lack of control for antibiotic use and stewardship program [2, 3].

In the past few decades, antimicrobial drugs have saved many lives and reduced the grief of many million people globally [3]. However, the extraordinary benefits of antimicrobials in reducing morbidity and mortality have been challenged by the emergence of drug resistant bacteria. The recent emergence and spread of these resistant bacteria have become a serious public health concern [4]. Especially, the spread of such bacteria in resource limited countries would have devastating consequences considering the health infrastructure, antibiotic options available and over all resource constraints observed in such countries [5].

In recent years, high dissemination of ESBL producing, carbapenem, and methicillin resistant bacteria are observed worldwide [6, 7]. It is described that the problem of ESBL-producing organisms is more intense in developing countries [8]. However, the magnitude of the problem is still probably underestimated due to inadequate or ineffective detection in some clinical settings [3, 7, 9]. ESBLs are a group of plasmid-mediated, diverse, complex and rapidly evolving enzymes which are capable of hydrolyzing penicillin’s, broad-spectrum cephalosporin’s and monobactam’s [10]. Accordingly, ESBL enzyme producing bacteria have a capacity to resist the action of penicillin’s, broad-spectrum cephalosporin’s and monobactam’s [11]. Furthermore, there is an evidence indicating that most of ESBL producing bacteria are also resistant for carbapenem antibiotics [12, 13]. ESBLs production is most commonly seen among Gram negative bacteria including *Escherichia coli, Klebsiella pneumoniae, Proteus mirabilis and Pseudomonas aeruginosa* [14].

Infections resulting from antibiotic resistant bacteria are more difficult and, in some instances, impossible to treat with current available antibiotics. Such infections lead to higher morbidity and mortality, imposing huge healthcare cost [15, 16]. In recent years, varieties of bacteria are becoming resistant against two or more classes of antibiotics as a result of selective pressure or horizontal gene transfer. For instance, the magnitude of resistance seen among *E. coli*, *S. aureus*, *Klebsiella* species, *P. aeruginosa*, *A. baumannii,* and *Enterobacter* species is more threatening as these bacteria are the commonest etiologies for commonly observed hospital and community acquired infections [17, 18].

In Ethiopia, the patterns of antibiotic resistance among commonly seen bacterial etiologies have been described previously in different settings [19–21]. However, most of these studies did not address the magnitude of ESBL producing and carbapenem resistance patterns comprehensively. It is also known that bacterial antibiotic resistance is a dynamic process. Resistance patterns seen in the past might not be representing the current situation due to the strong correlation between efficiency of antibiotic use and antibiotic resistance. As a result, information about the current antibiotic resistance pattern of bacteria is very vital to understand the dynamic and trend of resistance.

Clinical characteristics of patients with HAIs at Jimma University Medical Center have recently been published. The incidence and overall prevalence of HAIs at the hospital were 28.15 per 1000 patient days and 19.41% respectively [22]. In the current study, we aimed to determine the MDR, XDR, PDR, ESBL mediated and carbapenem resistance patterns of bacteria isolated from patients with HAIs at the hospital.

## Methods and materials

Institution based cross-sectional study was carried out in all wards of JUMC from May, 2016 to September, 2016. Totally, 1015 patients were admitted, of these 197 patients had sign of healthcare associated infection during the study time and all were taken as study participants. Microbiological investigation was done for 192 participants suspected to have healthcare associated infection; no microbiological test was done for the other five cases due to inability to obtain proper specimen. Different clinical specimens (blood, urine, wound swab, pus, and sputum) were collected aseptically from the patients with signs of healthcare associated infection. Bacterial identification was performed by standard microbiological methods which are adopted from CLSI guideline.

### Phenotypic determination of antibiotic susceptibility patterns

Antibacterial susceptibility of Penicillin (10 μg), Oxacillin (1 μg), Gentamycin (10 μg), Chloramphenicol (30 μg), Tetracycline (30 μg) Erythromycin (15 μg), Trimethoprim-sulfamethoxazole (1.25 g), Clindamycin (2 μg), Cefoxitin (30 μg), Ciprofloxacin (5 μg), Nitrofurantoin (300 μg), Norfloxacin (10 μg), Ampicillin (10 μg), Amoxicillin-clavulanic acid (10 μg), Ceftriaxone (30 μg), Ceftazidime (30 μg), Cefepime (30 μg), and Meropenem (10 μg), (Oxoid, UK) were determined in vitro by Kirby-Bauer disk diffusion method following Clinical and Laboratory Standards Institute guidelines [23].

### ESBL and/or AmpC detection

The presence of an ESBL and/or AmpC was determined with Cefpodoxime (10 μg), Cefotaxime (30 μg), Cefepime (30 μg) and Ceftazidime (30 μg) containing antibiotic discs (Mast Group, UK) by disc diffusion confirmation test. After the discs were inserted on inoculated plates, then they were incubated at 35–37 °C for 18–24 h aerobically. Finally, zones of inhibition were read and recorded on excel sheet. The data from the excel sheet was transported to Mast group ESBL/AmpC and CARBA plus calculator spreadsheet (Mast group, UK) and reported as negative or positive for ESBL or/and AmpC and finally the results were recorded.

The results were registered as resistant, intermediate and susceptible; but for the sake of analysis intermediate and resistant isolates were grouped together as resistant. Classification of MDR, XDR and PDR were carried out according to Magiorakos et al, definitions [4]. All the antibiotic disks were from Oxoid (Oxoid, UK) and Mast discs (Mast group, UK). The inhibition zone diameter was measured using caliper and recorded on excel sheet.

### Data quality control

Standard operating procedures (SOPs) were strictly followed while we did all bacteriological procedures starting from sample collection, isolation, identification and antibiotic susceptibility testing. Susceptible American Type Culture Collection (ATCC) 25,922 *E. coli* and ATCC 25923 *Staphylococcus aureus* were used as control strains and the test results were only accepted when the inhibition zone diameters of the above mentioned control strains were within performance ranges as described by CLSI [23]. ESBL positive ATCC 700603 *Klebsiella pneumoniae* and both ESBL and carbapenemase negative *E. coli* ATCC 25922 control strains were used in this study as positive and negative control respectively. To standardize the inoculum density of bacterial suspension for a susceptibility test, 0.5 McFarland standards, which is comparable with the approximate number of bacterial suspension (1.0 × 10^8^ to 2.0x10^8^bacteria/mL), was used [23].

### Data analysis and statistical tests

Data were double entered to Epi Data version 3.1 and transferred to SPSS version 20 and Microsoft Excel software for analysis and the results were presented as tables, pie-charts and graphs. *P*-values < 0.05 were considered as statistically significant.

### Ethical consideration

The study was approved by the Institutional Review Board of Institute of Health, Jimma University. Informed written consent was also obtained from participants and/or guardians after explaining the objective of the study. All the laboratory results were communicated as early as possible with the treating physicians for better management of the patients.

## Result

### Socio-demography and background information of the participants

From 1015 patients who were enrolled in the study; only 197 admitted patients had developed sign of healthcare associated infection with in the study time. Of these, 118 (59.9%) patients had culture confirmed healthcare associated infections. Sociodemographic and clinical characteristics of study participants have recently been published. The incidence and overall prevalence of HAIs at the hospital were 28.15 per 1000 patient days and 19.41% respectively [22].

### Isolation rate

**Fig. 1:**
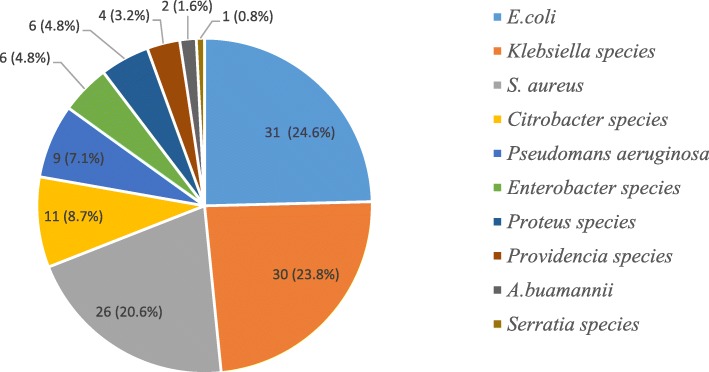
Proportions of isolated bacterial pathogens

Totally 240 clinical samples were obtained from 192 patients who were clinically diagnosed with healthcare associated infection. The most common sources of specimen were urine (55%) followed by wound swab/pus (24.2%), blood (15%), and sputum (5.8%). A total of 126 bacterial pathogens were isolated from 118 patient samples. A single organism was isolated from 110 (93.2%) patient samples, and two organisms were isolated from 8 (6.8%) patient samples who had been admitted to ICU. The overall culture positivity rate of participants was 118/192(61.5%). Most commonly isolated bacteria were *E. coli* 31(24.6%), *Klebsiella* species 30(23.8%) and *S. aureus* 26 (20.6%) (Fig. 1).

### Drug resistance patterns of isolates to different classes of antibiotics

Antibiotic resistance patterns of the isolated pathogen of nosocomial origin are shown in Table 1. Half of *S. aureus* isolates were resistant to gentamicin 50.0% (13/26); and 53.84% (14/26) and 57.7% (15/26) of the isolates were resistant to methicillin /cefoxitin/oxacillin and ceftriaxone/chloramphenicol in vitro respectively; and all of *S. aureus* isolates were resistant against penicillin (Table 1). From a total of 26 *S. aureus* isolates, 3(11.5%), 10(38.5%) 10 (38.5%) and 3(11.6%) were MoDR, MDR, XDR and PDR respectively (Table 2).

**Table 1:** Frequency of antimicrobial resistant bacterial isolates for selected antimicrobial classes

Antibiotic classes	Antibiotics	*S. aureus* (*n* = 26)	*E. coli* (*n* = 31)	*Klebsiella* species (*n* = 30)	*Citrobacter* species (*n* = 11)	*P. aeruginosa* (*n* = 9)	*Enterobacter* species (*n* = 6)	*Proteus* species (*n* = 6)	*Providencia* species (*n* = 4)	A. *buamannii* (*n* = 2)	*Serratia* species (*n* = 1)
Penecillins 3rd and 4th generation cephalosporins	Penicillin	26	–	–	–	–	–	–	–	–	–
	Ampicillin	–	29	30	11	9	6	5	4	2	1
	Ceftriaxone	15	15	16	7	9	4	4	2	2	1
	Ceftazidime	–	16	17	8	8	5	4	2	2	1
	Cefepime	–	14	15	6	5	2	4	2	2	1
Anti-staphylococcal β-lactams	Oxacillin	14	–	–	–	–	–	–	–	–	–
Cephamycins	Cefoxitin	14	19	23	6	8	6	4	1	2	1
Aminoglycosides	Gentamycin	13	22	21	7	8	3	3	1	2	1
Phenicols	Chloramphenicol	15	19	20	8	9	1	3	3	2	1
Macrolides	Erythromycin	19	–	–	–	–	–	–	–	–	–
Lincosamides	Clindamycin	17	–	–	–	–	–	–	–	–	–
Tetracycline	Tetracycline	17	29	27	7	9	4	6	4	2	1
Folate pathway inhibitors	Trimethoprim-sulfamethoxazole	19	28	24	9	4	2	6	3	2	1
Fluoroquinolones	Ciprofloxacin	16	14	12	6	6	3	3	2	1	1
Carbapenems	Meropenem	–	5	9	2	4	1	0	1	2	1
Penecillins + β-lactamase inhibitors	Amoxicillin-clavulanic acid	–	28	29	9	9	6	6	4	2	1

**Table 2:** Frequency distribution of MultiS, MoDR, MDR, XDR, and PDR pattern of isolated bacteria

Isolated organisms	Total	MultiS	MoDR	MDR	XDR	PDR
*S. aureus*	26	0	3	10	10	3
*E. coli*	31	0	3	11	10	7
*Klebsiella* species	30	4	2	9	13	2
*Citrobacter* species	11	0	0	2	4	5
*Enterobacter* species	6	0	0	2	3	1
*Proteus* species	6	0	0	2	4	0
*Providencia* species	4	0	0	0	3	1
*Pseudomonas aeruginosa*	9	0	0	2	4	3
*Acinetobacter baumannii*	2	0	0	0	1	1
*Serratia* species	1	0	0	0	0	1
Total	126	4	8	38	52	24

MultiS, susceptible to all antibiotic classes; MoDR, resistant to single antibiotic class; MDR, resistant to at least one agent in three or more antimicrobial categories; XDR, resistant to at least one agent in all but two or fewer antimicrobial categories (i.e. bacterial isolates remain susceptible to only one or two categories); PDR, resistant to all antibiotic classes. Source: Based on definitions by Magiorakos et al. [4]

From Gram negative bacteria, *E. coli* and *Klebsiella* species were the most frequent isolates. More than 90% of *E. coli* isolates were resistant against ampicillin, tetracycline and trimethoprim-sulfamethoxazole (Table 1). Conversely, only 16.1% of *E.coli* isolates were resistant against meropenem. Likewise, the resistance rate of *Klebsiella* species were 100% for ampicillin, 90% for tetracycline, 80% for trimethoprim-sulfamethoxazole, 40% for ciprofloxacin and 30% for meropenem (Table 1).

### Classification of isolates based on their drug resistance pattern

As shown in Table 2, among 126 bacterial isolates, 38 (30.2%), 52 (41.3%), and 24 (19%) were MDR, XDR, and PDR respectively. Eight of the isolates were resistant to a single antimicrobial class and only four *Klebsiella* isolates were susceptible to all classes of the antimicrobials. The predominant isolates (*E. coli*, *Klebsiella* species and *S. aureus)* showed very high antimicrobial resistance patterns. The overall MDR rate of the isolated bacteria was 30.16%. All bacteria isolated from ICU and pediatrics wards, 87.5% of bacteria isolated from Gynecology and obstetrics wards, 88% of bacteria isolated from Medical wards and 85.7% of bacteria isolated from surgical wards were MDR. The overall prevalence of PDR among all isolates was 19.0%. *Citrobacter species* (45.4%) and *Pseudomonas aeruginosa* (33.3%) have shown high pandrug resistance rate. On the other hand, *Klebsiella* species (6.6%) and *S.aureus* (11.5%) have shown the least PDR rate. *E.coli* (22.6%) and *Enterobacter species* (16.7%) have also shown a moderate PDR rate.

### Prevalence of ESBL, AmpC, and Carbapenemase producing isolates

Of the 1 hundred isolated gram-negative rods, 36 and 7% were positive for extended spectrum beta-lactamase (ESBL) and AmpC respectively. Eight percent of the isolates were positive for both extended spectrum beta-lactamase (ESBL) and AmpC. With regard to the proportion of carbapenemase producing isolates, 25% of gram negative isolates have shown carbapenem resistance (Table 3). To be precise, 16.1% of *E.coli* and 30.0% of *Klebsiella* species were carbapenem resistant isolates (Table 3).

**Table 3:** Prevalence of ESBL, AmpC, and Carbapenem resistant isolates of gram negative rods

Isolated organisms	Total	ESBL & AmpC producing isolates				Carbapenemase resistance	
		Not ESBL & AmpC, N (%)	ESBL, N (%)	AmpC, N (%)	ESBL & AmpC, N (%)	Yes, N (%)	No, N (%)
*E. coli*	31	12(38.7)	14(45.2)	3 (9.7)	2 (6.5)	5 (16.1)	26 (83.9)
*Klebsiella* species	30	14 (46.7)	13 (43.3)	2 (6.7)	1 (3.3)	9 (30.0)	21 (70.0)
*Citrobacter* species	11	5	4	0	2	2	9
*Enterobacter* species	6	2	3	0	1	1	5
*Proteus* species	6	5	1	0	0	0	6
*Providencia* species	4	1	2	1	0	1	3
*Pseudomonas aeruginosa*	9	5	1	1	2	4	5
*Acinetobacter baumannii*	2	2	0	0	0	2	0
*Serratia* species	1	1	0	0	0	1	0
Total	100	49	36	7	8	25	75

### Antimicrobial resistance pattern and impact on clinical outcome

Of 118 patients with culture confirmed healthcare associated infection, 13 patients (11.02%) died and all of the isolated microorganisms from these 13 patients were multidrug resistant (MDR) as shown in Table 4. The mean hospital stays of the patients infected with MDR bacteria were 15.4 ± 9.6 days (range 3–49 days). There is statistically significant association between mean duration of stay and infection with MDR bacteria (Table 4).

**Table 4:** Antimicrobial resistance and associated factors

Variable	Non MDR (*N* = 12) (%)	MDR (*N* = 106) (%)	*P*
History of treatment^a^			
No	9 (75.0)	85 (80.19)	
Yes	3 (25.0)	21 (19.81)	0.672
Patient outcome			
Progress	12 (100)	93 (87.74)	
Died	0	13 (12.26)	0.198
Duration of stay in Hospital			
<=15 days	8 (66.6)	25 (23.58)	
> 15 days	4 (33.4)	81 (76.42)	0.002

Non-MDR: susceptible to all antibiotic classes/resistant to one/two antibiotic classes; MDR: resistant to at least one agent in three or more antimicrobial categories Magiorakos et al. [4]

^a^ taking antibiotics in the last 3 months of the study period

## Discussion

The overall rate of MDR, XDR and PDR bacterial isolates from JUMC were found to be 30.16, 41.27 and 19.0% respectively. Furthermore, the observed MDR rate is significantly associated with prolonged hospital stay and all patients, who died, were infected with MDR bacterial species (even if, it is not statistically significant). On the other hand, the observed XDR and PDR rate at the hospital indicates that the problem of AMR is increasing at an alarming rate and pathogenic bacteria that circulate in JUMC are becoming more resistant to all available antibiotics. The occurrence of PDR pathogenic bacteria would also have huge potential threat and implications for patient care in the hospital and the community at large. As we are living in the era of very connected world, it is highly likely for these PDR bacteria to be disseminated to other parts of Ethiopia and other parts of the world as well.

To the best of our knowledge, there is no previous report from Ethiopia on the rate of XDR and PDR pathogenic bacteria to compare with this result. It is possible to list some reasons which might have contributed for this observed high XDR and PDR rate. The first reason might be associated with lack of AMR surveillance and stewardship program at JUMC and in Ethiopia in general. There is enough evidence that indicates AMR surveillance and stewardship program helps to understand the pattern of resistance and improve the utilization of antibiotics to prevent occurrence of antibiotic resistance.

The second reason might be associated with lack of comprehensive national antibiotic policies and problems in implementations of policies. In Ethiopia, there is no clear antibiotic policy and controlling mechanism about antibiotic usage. It is a common practice in Ethiopia to buy any antibiotic from private drug vendors and pharmacies without any prescription. This might have contributed for emergence and dissemination of antibiotic resistant bacteria at different settings. The third reason might be associated with lack of system to assess the quality and reliability of imported antibiotics in Ethiopia. For instance, one previous study which assessed the quality of anti-tuberculosis drugs in Ethiopia in 2013 has indicated that around 17% of anti TB drugs were fake drugs [24]. It is easy to imagine the role of these fake drugs on anti TB drug resistance. Likewise, though there is no previous research done in Ethiopia to assess the quality of antibiotics dispensed in private and government pharmacies, it is highly likely that some of them might be sub-standard, given that substantial proportion of the antibiotics in private pharmacies are supplied through unknown routes [25, 26].

The emergence of ESBL producing gram negative rods have become a rising concern in the developing world [27]. In this study, phenotypically, the most common ESBL producing microorganisms were *E. coli* and *Klebsiella* species which are 51.6% (16/31) and 46.7% (14/30) respectively; which is comparable with the studies done in Nigeria, Nepal and New Zealand in which ESBL producing *Enterobacteriaceae* were 44.3, 43.7 and 38.0% respectively [14, 28, 29]. However, the other studies done in India and Nepal showed that 30.18 and 18.4% of *Klebsiella pneumoniae* produce ESBL respectively which is lower than our report [30, 31]. Even though the prevalence of ESBLs is not well documented, in many parts of the world 10–40% of strains of *Escherichia coli* and *Klebsiella pneumoniae* are estimated to produce ESBLs [27]. High proportion of ESBL producing isolates was documented in the current study which might be due to the fact that our study participants were all hospitalized; since hospitalization was identified as the strongest independent risk factor to express ESBL [32].

Regarding to carbapenem resistance, 19 (21.4%), 4 (44.4%) and 2 (100%) of the *Enterobacteriaceae*, *P. aeruginosa* and *A. buamannii* were carbapenem resistant respectively which are found in the priority one list according to WHO classification [33]. In addition to that, 53.8% of the other commonly isolated *S. aureus* were methicillin resistant which also needs high attention. Therefore, high attention should be given to these pathogens which are considered as priority one and two according to WHO [33]. To compare with other similar studies, the rate of carbapenem resistance among *E.coli* (16.1%) and *Klebsiella* species (30%) is consistent with multinational study done in Europe [34]. In contrary, 25% carbapenem resistance rate observed in this study is lower than a report from Brazil which was 100% [35]. This could be explained by the difference in utilization of carbapenem antibiotics to treat different infections in the respective setups [36, 37]. The observed high carbapenem resistance rate can also be due to prescription of antibiotics without the knowledge of their susceptibility pattern, or introduction and dissemination of carbapenem resistant bacteria strains from other areas with high resistance rate might also be possible as JUMC is frequently visited by different European, Chinese and Korean nationalities due to different collaborative researches, training and service activities.

As reported by other studies, meropenem was the most effective antibiotic against most gram-negative rods [38]. To control high rate of antibiotic resistant isolates coordinated and urgent action is needed to prevent the development of drug resistance in the setting. Surveillance on antibiotic resistance will also be most useful to decide the correct empirical treatment and will help to control and prevent infections caused by resistant pathogens. Furthermore, our data suggest that the most effective antibiotics for gram-negative bacilli in vitro are meropenem followed by cefepime and for gram-positive organisms less resistance was observed against gentamycin.

## Conclusion

In this study, high antimicrobial resistance rate was demonstrated. The observed high PDR, ESBL and carbapenem resistance rate is worrisome. Coordinated effort is needed from all stakeholders working in health system in Ethiopia to tackle this important public health problem. An immediate action should be taken at the hospital to start antibiotic stewardship program to reduce the observed antibiotic resistance and prevent further complications.

## Abbreviations

HAI: Health care associated infection
MDR: Resistant to at least one agent in three or more antimicrobial categories
MoDR: Resistant to single antibiotic class
MultiS: Susceptible to all antibiotic classes
PDR: Resistant to all antibiotic classes
XDR: Resistant to at least one agent in all but two or fewer antimicrobial categories (i.e. bacterial isolates remain susceptible to only one or two categories)
